# Tweeting about alcohol: Exploring differences in Twitter sentiment during the onset of the COVID-19 pandemic

**DOI:** 10.1371/journal.pone.0276863

**Published:** 2022-11-03

**Authors:** Jeffrey A. Stone, Nicole C. Ryerson

**Affiliations:** 1 Department of Information Sciences and Technology, Penn State University, Center Valley, PA, United States of America; 2 Department of Psychology, Penn State University, Center Valley, PA, United States of America; Chiang Mai University, THAILAND

## Abstract

This study explores pandemic-related changes in Twitter communication by examining differences in emotional, psychological and social sentiment between alcohol-related tweets and a random sample of non-alcohol tweets during the onset of the COVID-19 pandemic. Two equivalent size sets of English-language, COVID-specific tweets posted between February 1^st^ and April 20^th^, 2020 are examined. The first set includes 1.5 million tweets containing alcohol-related keywords, while the second set does not contain such references. LIWC software analyzed the tweets for sentiment factors. ANCOVAs were used to determine whether language use significantly differed between the sets, considering differences in the pandemic period (before or after the pandemic declaration) while controlling for the number of tweets. The study found that tweets in the 40 days after March 11, 2020 contained more authentic language, more affiliation-oriented language, and exhibited more positive emotion than tweets in the 40 days pre-declaration. Alcohol-related status was a significant factor only when tweets contained personal concerns, regardless of pandemic period. Authenticity levels increased significantly in alcohol-related tweets post-declaration. The findings suggest alcohol may play a lesser role in the expression of psychological, social, and emotional sentiment than the pandemic period, but interaction between authentic language and alcohol references may reflect an increased use of alcohol for coping.

## Introduction

The COVID-19 pandemic has resulted in a plethora of societal changes, including changes in social patterns (distancing, quarantining, and restricted activities), economic patterns (increased online commerce, business shutdowns), and in how work and learning are performed (increased use of remote work and videoconferencing tools). Initial research has found vast emotional, psychological, and social impacts as a result of these abrupt and severe changes to daily life brought about by the onset of the pandemic. Specifically, research has demonstrated increased prevalence of mental health issues [1], increased social isolation and associated effects of loneliness [2], and an overall increase in negative emotions with a decrease in positive emotions [3].

Increased emotional and psychological stress has been shown to increase chances of substance abuse, including alcohol. General life stress and stress related to catastrophic events can lead to an increase in alcohol consumption and an increased risk for alcohol use disorders [4]. The onset of the pandemic arguably introduced both types of stress in a chronic fashion for most individuals. As people sought coping mechanisms for the stress brought on by the pandemic, social media provided an easily accessible outlet for individuals to share information about their concerns, their personal well-being and their daily activities. Social media facilitates the creation and maintenance of social connections that can be especially important during periods of relative isolation. Examining user posts from the social media microblog Twitter can provide insight into coping behaviors as well as the emotional and psychological state of users; prior research has shown that social media offers a meaningful data source for exploring emotional, psychological, and cognitive constructs [5]. There is currently a lack of research exploring whether the tweets of users who post with alcohol-related language exhibit different levels of emotional, psychological, and social sentiment than tweets which include no alcohol-related references. Assessing the linguistic content of tweets can provide insight into whether alcohol may act as a coping mechanism for COVID-related stressors, and whether and how alcohol references are combined with increased references to emotional states, social affiliation, and personal concerns.

The purpose of this study was to identify comparative differences in emotional, psychological and social sentiment between alcohol-related tweets and a random sample of non-alcohol-related tweets through the initial onset of the pandemic (from February through April, 2020). Identifying these differences can shed light on how the onset of the pandemic influenced Twitter communication, specifically emotional, psychological, and social references and sentiment. It was expected that alcohol-related tweets would exhibit increased levels of language reflecting honesty and confidence than unrelated tweets in the post-pandemic period (post-March 11), indicating a potential use of alcohol as coping mechanism for pandemic-induced stress. It was further expected that COVID-related social isolation would cause alcohol-related tweets to include more emotional and affiliation-oriented language than unrelated tweets during this same period. Finally, it was anticipated that alcohol-related tweets would include more references to personal concerns (e.g., faith, money, home life, death), given the increased likelihood of occupational and financial uncertainty during the early days of the pandemic.

## Background

### Change, stress, and impacts due to COVID-19

Between late 2019, when the COVID-19 virus was first confirmed in Wuhan China, and March 11^th^, 2020, when the virus was declared a pandemic by the World Health Organization, COVID-19 rapidly spread to over 114 countries. The initial onset of the pandemic resulted in world-wide government mandates intended to slow the spread of the virus (e.g., travel restrictions, mask requirements, business closures). These changes disrupted daily life for people worldwide, and with these abrupt changes came many sources of stress with far-reaching consequences. Looking back on the initial impact of the pandemic can provide insight into its continuing effects and may provide some foreshadowing of the issues that arise as individuals begin to phase back into normal routines.

Mental health issues quickly increased during the early days of the pandemic (see [1]) including a global rise in anxiety and depression (see [6]). Research has pointed to loneliness as being a major contributor to the rise in depression, anxiety and PTSD symptomology with family support serving as a protective factor against these negative mental health outcomes [7]. These results confirm the long-established role that social affiliation plays in mental health outcomes [8] and further highlights the significant impact of the pandemic on social processes. There is also evidence for increased stress and PTSD symptoms relating to fears surrounding the virus’s health impacts (i.e., contracting or spreading the virus, the virus mortality rate) [9]. Many of these mental health outcomes have contributed to an overall increase in negative emotions [3].

Beyond mental health concerns, the initial onset of the pandemic also resulted in a range of personal concerns. Internationally, most individuals experienced a loss of financial opportunities due to a complete loss or reduction in employment hours [10]. In the United States, the onset of the pandemic resulted in the highest rate of unemployment since the Great Depression [11]. For many of those who remained employed, there was a shift to working remotely from home. Home life dynamics were also impacted by the shift to remote learning for many students. Furthermore, in the United States alone over 2.2 million young adults reported moving back home with a parent or grandparent between March and April 2020 [12]. Finally, many families have reported disruptions to their ability to access essential resources [13]. Taken together this research provides a snapshot of how personal concerns have been shifted by the onset of the pandemic.

Each of these identified impacts has inarguably led to an increase in stress for most of the world’s population (see [14]). As a result of this increase in stress, individuals have likely either adopted new coping strategies or may have relied more heavily on already established coping strategies, including substance use. And in fact, previous research has demonstrated the use of alcohol as a coping mechanism during community wide disruptions that may be used as a comparison for the current pandemic.

### Alcohol as potential coping mechanism

As previously demonstrated, the onset of the global pandemic has resulted in an increase in general life stress as well as stress related to the catastrophic effects of the pandemic. Previous research has demonstrated that exposure to both types of stress can result in increases in alcohol consumption and an increased risk for alcohol use disorders [4]. Alcohol has long been implicated as a substance that is commonly used to self-medicate in response to distress [15], as a means of reducing tension [16], and as a mechanism for regulating negative emotions [17]. However, it should be noted that not all individuals turn to alcohol as a means of coping. For example, individuals are more likely to turn to alcohol as a coping strategy when other strategies are unavailable (see [18]).

Some coping strategies that can serve as a buffer against alcohol consumption include the use of adaptive problem-focused strategies [19, 20] and seeking social support [19]. Whereas individuals using avoidant coping strategies are more likely to use alcohol as a means of coping with stress [21]. The implications of this previous research (that alcohol is more commonly used to cope when access to alternative coping strategies is restricted and that active coping strategies as well as social support strategies can serve as a buffer) is especially troubling in the current pandemic. Specifically, at the onset of the pandemic restrictions were put in place that would inherently limit access to alternative activities as well as social networks. However, individuals still had access to alcohol, and in fact alcohol sales increased both online and in stores [22].

Prior research on events resulting in community-wide disruptions have also pointed toward alcohol use as a common coping strategy among those impacted. For example, research has shown that alcohol consumption increases following economic crises [23] as well as exposure to natural and man-made disasters [24]. This research can be directly compared to the current community-wide disruptions resulting from the COVID-19 pandemic. It is also worth noting that declines in psychological health are also related to increased alcohol consumption [25], potentially as a means of coping [26]. This is concerning when considering an announcement made by the World Health Organization indicating the likely decline in mental health worldwide as a result of the pandemic [27].

Assessing alcohol references within social media may provide an efficient method for capturing the use of alcohol as a means of coping with the effects of the pandemic. Previous research has effectively demonstrated the use of social media content to reflect alcohol consumption behaviors [28]. Beyond simply assessing the use of alcohol, analyzing the content of alcohol related tweets can highlights the emotional, psychological and social sentiment related to these coping strategies.

### Social media as indicators of well-being

The analysis of social media posts to assess the well-being of populations has been accepted as a viable alternative to more traditional survey-based research [29]. Twitter has become an attractive source of real-time information on moods, emotions, and other psychological indicators. The brevity of communication–at most 280 characters per “tweet”—coupled with its relative ease of use allows users to share not only thoughts, feelings, and real-time activities, but also information from others they find compelling (“retweeting”). Twitter has become a popular platform for social media communication; some estimates posit that 500 million tweets a day are posted to Twitter from over 150 million active daily users [30].

The constantly accessible yet asynchronous nature of social media provides opportunities for self-disclosure and relationship-building that may be more palatable than more traditional, synchronous communication methods. Social media provides a comparatively easy mechanism for building a large social network and associated social capital through the reduction of spatial and temporal constraints [31]. Prior research has shown that maintaining a social media network of “friends” and contacts can contribute to enhanced well-being and social satisfaction [32]. However, some have argued that the specific social media activities undertaken by users ultimately determines its efficacy at enhancing and maintaining social relationships [33].

Tweets referencing personal situations, emotions, and struggles can be expected during crisis events, including pandemics [34]. Prior research on crisis events such as 9/11 [35] found that people increased their use of first-person pronouns in response to the event, especially plural pronouns (e.g., “we”, “us”, “our”). Similar findings were reported in responses to smaller, tragic events [36]. These findings may indicate that such events lead to an increased sense of togetherness or group cohesiveness [37] which can assist in the coping process. For the COVID-19 pandemic, early research has indicated an increase in social media interaction and engagement with COVID-related posts [38], including the sharing of sometimes questionable information on social media, even with altruistic intentions [39]. References to sociality and social groups—i.e., affiliation references–have been shown to increase in the short-term after a crisis event but return to a normal level after as few as two weeks post-event [35]. Given the extended nature of the COVID-19 pandemic, it is reasonable to assume that increased references to affiliation will occur, and that these references may persist for a longer time period.

Prior research into the impact of catastrophic events on emotional tone often find at least a temporary increase in negative emotionality [40] though positive emotions can act as a "shield" of sorts to protect individuals’ mental health after a crisis event has occurred [35, 41]. Given that it is common for negative emotion words to be used when writing about a negative event [5], it is sometimes challenging to separate the description of such events from personal expressions of emotional response. Given the increased stress associated with the pandemic, it also is reasonable to expect that Twitter posts would see an increase not only in negative emotionality but also in references to personal concerns (e.g., work, money, health, religion, death). The increased use of telecommuting, coupled with government-mandated lockdowns, meant that work-related stress was likely intermingled with stress related to personal, home-based concerns. The increased stress can manifest itself in an increased use of self-referential language (e.g., “I”), which research has shown may inhibit the ability of individuals to avoid negative emotionality and therefore may result in inappropriate or detrimental behaviors and associated health outcomes, including abuse of alcohol [42, 43]. Increased stress can also lead to the sharing of alcohol-related events through social media outlets. For example, preliminary research by Ryerson and Stone [44] suggests that references to alcohol within COVID-related tweets increased significantly following the global pandemic declaration.

### LIWC as psychological and emotional assessment

Assessing the content of tweets can provide insight into the emotional, psychological, and behavioral state of the communicator. The Linguistic Inquiry and Word Count (LIWC) software provides automated analysis of text-based data for pre-defined affective, cognitive, structural, and behavioral constructs using approximately 80 predefined linguistic categories (see [45] for a full description). These categories reflect emotional, cognitive, and structural constructs, including sadness, anger, certainty, and time orientation, and categories have previously been combined by researchers to represent constructs such as honesty, leadership, and cognitive complexity. LIWC analyzes a body of text and generates a numeric measure for each linguistic category. These output measures represent the percentage of words in the text that match the predefined dictionaries for that category. In addition, LIWC provides four summary variables for *Analytical Thinking*, *Emotional Tone*, *Clout*, and *Authenticity*. Rather than specific predefined categories, these summary variables represent composites of select others on a 0–100 scale [46]. A wealth of prior research has shown the correlation between LIWC output measures and psychological, social, and emotional factors, including in regard to social media data (e.g. [29, 47–49]). The validity and reliability of LIWC has also been supported by a diverse group of studies [5].

### Purpose and research questions

This study explores changes in Twitter communication surrounding the onset of the COVID-19 pandemic. Specifically, this study used LIWC software to identify comparative differences in emotional, psychological and social sentiment between a set of alcohol-related tweets and a random sample of non-alcohol-related tweets from February through April, 2020. This study focused on linguistic analysis of tweets to answer the following research questions:

RQ_1_: Do tweets containing alcohol-related references exhibit significant differences in the level of honesty and confidence when compared to non-alcohol tweets, accounting for the pandemic period (pre- and post-)?
RQ_2_: Do tweets containing alcohol-related references exhibit significant differences in emotional tone when compared to non-alcohol tweets, accounting for the pandemic period (pre- and post-)?
RQ_3_: Do tweets containing alcohol-related references exhibit significant differences in the use of affiliation-oriented language when compared to non-alcohol tweets, accounting for the pandemic period (pre- and post-)?
RQ_4_: Do tweets containing alcohol-related references exhibit significant differences in the use of language related to personal concerns when compared to non-alcohol tweets, accounting for the pandemic period (pre- and post-)?

This study builds on the prior work of [44, 50, 51], and others to explore changes and patterns in communication and sentiment in social media use during the COVID-19 pandemic.

## Methodology

### Sampling method

Tweets were collected for the period February 1st, 2020 through April 20th, 2020. Tweet IDs were obtained from the GeoCoV19 dataset provided by CrisisNLP (https://crisisnlp.qcri.org/covid19). The GeoCov19 dataset [52] includes the IDs of over 500 million international tweets containing one or more of 800 COVID-19 related hashtags and keywords. The Hydrator application from Documenting the Now (http://github.com/DocNow/hydrator) was used to download the tweets of the available IDs. Some IDs were no longer accessible, but 84.85% of the provided IDs were successfully downloaded. Custom Python scripts were then used to convert the associated tweets into a format suitable for analysis. The resultant dataset included 242,550,744 English-language tweets (Per Day Mean = 3,031,884.30, SD = 2,019,969.53). Additional custom Python scripts were used as a filter to extract tweets containing one or more of 60 alcohol keywords taken from the codebook established by Alhabash et al. [53]. The final dataset included 1,523,420 tweets with at least one alcohol keyword (0.63% of the sample; Per Day Mean = 19,042.75, SD = 13,630.77) and 1,629,245 total alcohol keyword occurrences (0.67%, Per Day Mean = 20,365.56, SD = 14,565.09). All data collection and analysis complied with the Twitter terms and conditions.

To facilitate comparisons between tweets containing alcohol references and those tweets without such references, a random sample of tweets which did not involve any alcohol-related keywords was also taken from the original dataset of English-language, COVID-specific tweets. This random sample dataset included the same number of tweets as the sample of alcohol-related tweets, both in total (1,523,420) and on a per-day basis. Reservoir sampling was used to obtain the random sample (see [54]), given its relative ease of use and ability to obtain a specific random sample size from a large population.

### Data analysis procedures

A series of 2 (pandemic period: pre-pandemic vs. post-pandemic) x 2 (tweet type: alcohol-related or not alcohol-related) mixed Analysis of Covariance (ANCOVA) procedures were conducted to determine whether language use significantly differed between the set of alcohol-related tweets and the random sample of tweets without alcohol keywords, considering differences in the pandemic period while controlling for the overall number of tweets. The analyses were also used to determine if there were interactions between pandemic period and the alcohol-related status on language use. Given its long-standing use in similar analyses, LIWC was chosen as the tool to gather information on emotional, psychological, and social sentiment among the sample tweets. LIWC measures were computed for each day of the sample period, both for alcohol-related tweets and non-alcohol-related tweets.

The dependent variables for these analyses were operationalized from existing LIWC measures. Five ANCOVA models were created. The first three models utilized summary measures provided by the LIWC software–*Authenticity*, *Clout*, and *Emotional Tone–*while the final two models utilized composite measures constructed by the authors from more discrete LIWC measures. The decision to use a mix of existing summary measures and custom composites was made based on the preceding literature review, the general acceptance and validity of the existing LIWC measures, and the authors’ desire to identify comparative differences in emotional, psychological, and social sentiment at a more connected or abstract level rather than focusing on more discrete elements. A description and rationale for each of the dependent variables is provided in the following paragraphs.

The *Authenticity*, *Clout*, and *Emotional Tone* summary variables represent standardized composites using previously published algorithms and findings, and values exist on a 0–100 scale [45, 46]. The *Authenticity* measure considers the frequency of first-person singular and third-person pronouns, along with fewer exclusion words (e.g., without), and words expressing negative emotions [55]. In terms of social media use, honesty includes online self-disclosure and can be considered a positive coping strategy. Prior research has suggested that self disclosure can positively impact psychological well-being [56], including more honest self-disclosure [57]. For example, research by Zhen, Nan, and Pham [58] found that college students who use social media to disclose more personal information experienced less COVID-related stress. The *Authenticity* measure is used as a proxy for honesty (research question 1), given that a higher *Authenticity* value suggests "a more honest, personal, and disclosing text" [46] whereas a lower value suggests more guarded discourse.

The *Clout* measure is used as a proxy for confidence (research question 1), given that a higher *Clout* value suggests “the author is speaking from the perspective of high expertise and is confident" [46] whereas a lower value suggests a more tentative approach. Higher clout values are “indicated by more we-words and social words and fewer I-words, negations (e.g. no, not), and swear words” [59]. The *Clout* measure was used as a proxy for confidence given that the COVID-19 pandemic has been shown to impact self-confidence, among other mental health concerns [60]. Self-confidence is said to include not only things like optimism and self-affirmation, but also self-awareness and persistence (resilience); such attributes are especially vital in times of crisis, and a lack of these attributes can be indicators of more serious well-being concerns [61].

The *Emotional Tone* measure is used as the dependent variable for exploring research question 2; higher values represent a more positive emotional tone, whereas lower values represent a more negative tone. Values around the midpoint (50) represent ambivalence [46]. The *Emotional Tone* measure is constructed based on the number of negative (e.g. sadness) and positive words (e.g. happiness) in the corpus [35]. This measure was chosen due to its use in prior research, the aforementioned impact of crises events on emotional tone in social media posts, and its ability to provide a more general perspective of the emotional sentiment of posts. The combined roles of emotional support and self-confidence should also not be discounted; increases in emotional support can increase self-confidence, reducing stress and inhibiting maladaptive coping methods such as alcohol use [62].

The remaining two ANCOVA models use dependent measures constructed as composites from existing LIWC measures. The use of affiliation-oriented language (research question 3) is operationalized as the sum of four separate LIWC measures and is named *Affiliation Concerns*. The four LIWC measures used are affiliation (e.g. friend, ally), social processes (e.g. mate, talk), family references (e.g. daughter, son), and friend references (e.g. buddy, neighbor). The *Affiliation Concerns* composite measure was chosen due to the impact of the pandemic on social processes, the aforementioned relationship between social affiliation and mental health [2, 8] and disaster-replated coping [37], and prior research indicating the increased use of affiliation references during a crisis event [35].

Finally, a *Personal Concerns* measure (research question 4) was operationalized as the sum of six separate LIWC measures: work (e.g. job), leisure (e.g. cook, chat), home (e.g. kitchen, landlord), money (e.g. cash, owe), religion (e.g. church, faith), and death (e.g. coffin, kill). The *Personal Concerns* measure provides an indication of the impact that COVID-19 has had on potential stressors relating to employment, finance, faith, and health [11–13]. Descriptive statistics for each measure are listed in Table 1.

**Table 1:** Descriptive statistics for dependent variables.

	Alcohol Tweets		Non-Alcohol Tweets	
	Mean	SD	Mean	SD
*Emotional Tone*	24.863	12.614	26.146	6.493
*Authenticity*	8.288	5.790	7.794	1.332
*Clout*	66.878	8.587	68.413	3.035
*Affiliation Concerns*	8.799	2.320	8.801	1.252
*Personal Concerns*	7.497	1.401	5.585	0.410

## Results

### Differences in the use of authentic language

The ANCOVA results showed a significant main effect of the pandemic period (pre-pandemic vs. post-pandemic) on *Authenticity* (F(1, 77) = 12.880, p < 0.001, partial η^2^ = 0.143). No significant main effects were found for alcohol-related status (F(1, 77) = 2.098, p = 0.152). The covariate (total tweets) significantly influenced *Authenticity*, F(1, 77) = 7.136, p = 0.009, partial η^2^ = 0.085. The results show that users during the post-pandemic period used significantly more authentic language for both alcohol-related tweets (Mean = 9.769, SD = 7.032) and tweets unrelated to alcohol (Mean = 7.889, SD = 0.948) than during the pre-pandemic period (Mean = 6.808, SD = 3.738 and Mean = 7.699, SD = 1.635, respectively), controlling for the overall total number of tweets. Estimated marginal means are shown in Table 2.

**Table 2:** Estimated marginal means for pandemic period (main effects).

				95% Confidence Interval	
LIWC Measure	Period	Mean	SE	Lower Bound	Upper Bound
Authentic	Pre-Pandemic	5.891	0.677	4.543	7.238
	Post-Pandemic	10.191	0.677	8.844	11.539
Emotional Tone	Pre-Pandemic	20.940	1.417	18.120	23.761
	Post-Pandemic	30.069	1.417	27.248	32.889
Affiliation Concerns	Pre-Pandemic	8.207	0.279	7.562	8.762
	Post-Pandemic	9.392	0.279	8.837	9.948

**Fig 1:**
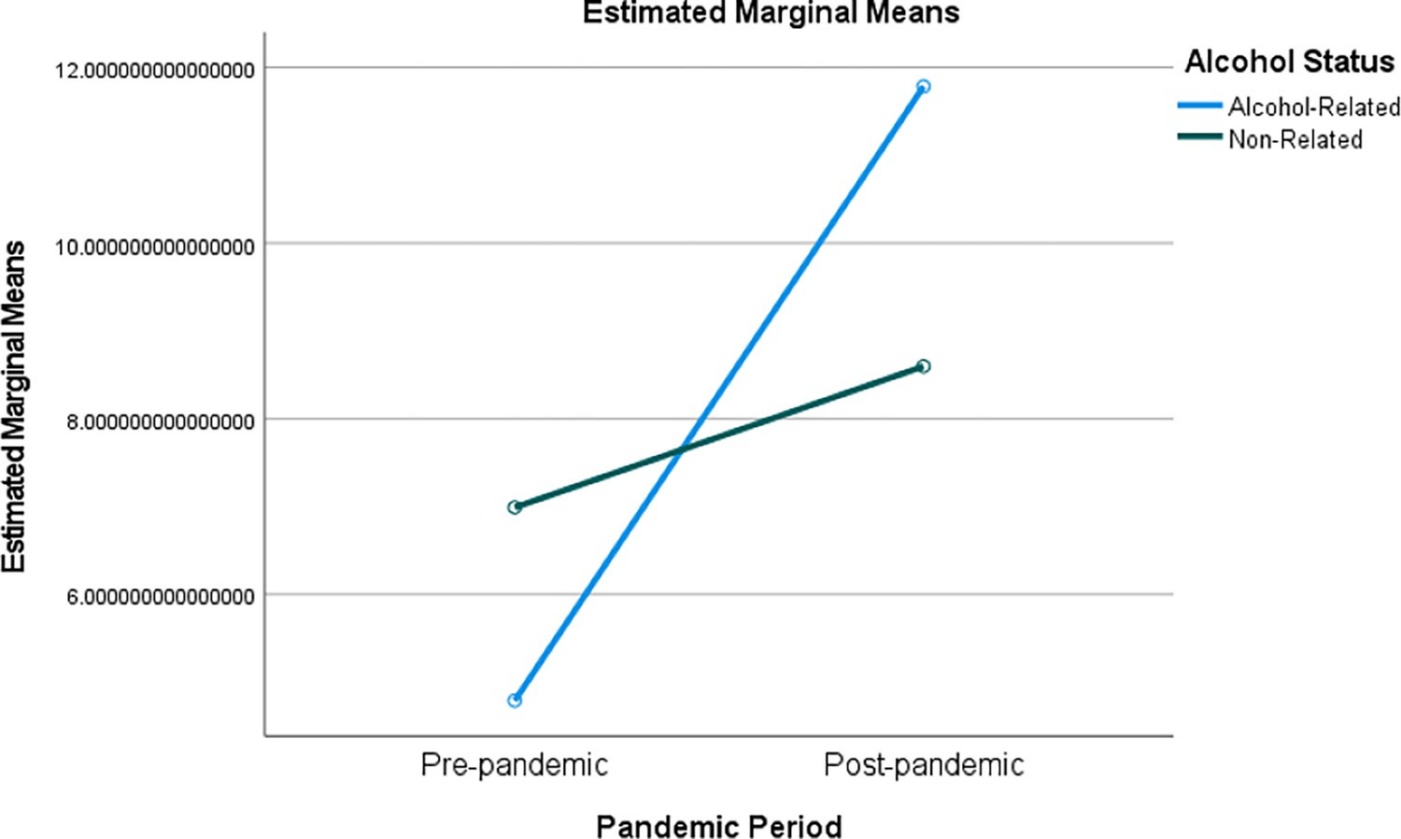
Estimated marginal means for alcohol status (pandemic interaction).

There was significant interaction between the alcohol status of tweet (alcohol-related or unrelated to alcohol) and the pandemic period on *Authenticity* (F(1, 77) = 4.904, p = 0.030, partial η^2^ = 0.060). Estimated marginal means are shown in Table 3. The results show that for authentic (honest) language, there is an inverse relationship between the pandemic periods and the alcohol status of the tweets. While the descriptive statistics showed that users during the post-pandemic period used significantly more authentic language for both alcohol-related tweets and tweets unrelated to alcohol than during the pre-pandemic period, the plot in Fig 1 shows that the gap between pre- and post-pandemic use of authentic language was much larger for alcohol-related tweets.

**Table 3:** Estimated marginal means for alcohol status and pandemic (interaction).

					95% Confidence Interval	
LIWC Measure	Pandemic Period	Alcohol Status	Mean	SE	Lower Bound	Upper Bound
Authenticity						
	Pre	Alcohol	4.791	1.329	2.144	7.437
		Non-Alcohol	6.991	0.305	6.382	7.599
	Post	Alcohol	11.785	1.329	9.139	14.432
		Non-Alcohol	8.597	0.305	7.989	9.206

In sum, these results show that tweets contained more authentic language (reflecting honest expression) in the post-pandemic period. While alcohol did not have a significant primary effect, the interaction between alcohol and the pandemic period suggests that authenticity levels increased significantly more in alcohol-related tweets during the post-pandemic period, perhaps reflecting an increased use of alcohol as a means of coping with the new reality.

### Differences in the use of confident language

The ANCOVA results did not show a significant main effect of the pandemic period on *Clout* (F(1, 77) = 0.055, p = 0.815). No significant main effects were found for alcohol-related status (F(1, 77) = 1.198, p = 0.170). The covariate (total tweets) significantly influenced *Clout*, F(1, 77) = 7.309, p = 0.008, partial η^2^ = 0.087. There was no significant interaction between the alcohol status of tweet (alcohol-related or unrelated to alcohol) and the pandemic period on *Clout* (F(1, 77) = 0.543, p = 0.463) nor between the alcohol status and the total number of tweets (F(1, 77) = 0.782, p = 0.379). The overall finding is that nether the pandemic period nor the alcohol-related status of the tweets significantly impacted the use of confidence-related language.

### Differences in emotional tone

The ANCOVA results showed a significant main effect of the pandemic period on *Emotional Tone* (F(1, 77) = 13.241, p < 0.001, partial η^2^ = 0.147). No significant main effects were found for alcohol-related status (F(1, 77) = 0.047, p = 0.830). The covariate (total tweets) did not significantly influence *Emotional Tone*, F(1, 77) = 1.749, p = 0.190. Therefore, ignoring other variables, emotional tone differed between alcohol- and non-alcohol-related tweets based on the pandemic period. Estimated marginal means are shown in Table 2.

The results show that tweets during the post-pandemic period had a significantly more positive emotional tone for both alcohol-related tweets (Mean = 31.240, SD = 11.600) and tweets unrelated to alcohol (Mean = 31.722, SD = 3.340) than during the pre-pandemic period (Mean = 18.486, SD = 10.215 and Mean = 20.570, SD = 3.236, respectively), controlling for the overall total number of tweets. There was no significant interaction between the alcohol status of tweet (alcohol-related or unrelated to alcohol) and the pandemic period on *Emotional Tone* (F(1, 77) = 0.207, p = 0.651) nor between the alcohol status and the total number of tweets (F(1, 77) = 0.018, p = 0.893).

In sum, these results show that tweets contained more positive emotional tone in the post-pandemic period for both alcohol-related tweets and unrelated tweets. Alcohol status was not a significant factor.

### Differences in the use of affiliation-oriented language

The ANCOVA results showed a significant main effect of the pandemic period on the use of language related to *Affiliation Concerns* (F(1, 77) = 5.760, p < 0.05, partial η^2^ = 0.070). No significant main effects were found for alcohol-related status (F(1, 77) = 0.510, p = 0.477). The covariate (total tweets) did not significantly influence *Affiliation Concerns*, F(1, 77) = 2.166, p = 0.145. Therefore, ignoring other variables, the use of affiliation-oriented language differed between alcohol- and non-alcohol-related tweets based on the pandemic period. Estimated marginal means are shown in Table 2.

The results show that tweets during the post-pandemic period had a significantly more affiliation-oriented language for both alcohol-related tweets (Mean = 9.608, SD = 1.698) and tweets unrelated to alcohol (Mean = 9.796, SD = 0.426) than during the pre-pandemic period (Mean = 7.990, SD = 2.369 and Mean = 7.806, SD = 0.983, respectively), controlling for the overall total number of tweets. There was no significant interaction between the alcohol status of tweet (alcohol-related or unrelated to alcohol) and the pandemic period on *Affiliation Concerns* (F(1, 77) = 0.071, p = 0.791) or between the alcohol status and the total number of tweets (F(1, 77) = 0.576, p = 0.450).

In sum, these results show that tweets contained more affiliation-oriented language in the post-pandemic period for both alcohol-related tweets and unrelated tweets. Alcohol status was again not a significant factor in the results.

### Differences in the use of language for personal concerns

The ANCOVA results did not show a significant main effect of the pandemic period on the use of language related to *Personal Concerns* (F(1, 77) = 0.116, p = 0.734). A significant main effect was found for alcohol-related status (F(1, 77) = 23.581, p < 0.001, partial η^2^ = 0.234). The covariate (total tweets) did not significantly influence *Personal Concerns*, F(1, 77) = 1.211, p = 0.274.

Therefore, ignoring other variables, language related to personal concerns significantly differed between alcohol- and non-alcohol-related tweets. Estimated marginal means are shown in Table 3. The results show that the use of language related to personal concerns–e.g. work, money, leisure, home, and religion–were significantly higher for tweets with alcohol references (Mean = 7.497, SD = 1.401) than for tweets without these references (Mean = 5.585, SD = 0.410). Estimated marginal means are shown in Table 4.

**Table 4:** Estimated marginal means for alcohol status (main effects).

				95% Confidence Interval	
LIWC Measure	Alcohol Status	Mean	SE	Lower Bound	Upper Bound
Personal Concerns	Alcohol-Related	7.497	0.156	7.186	7.808
	Not Related	5.585	0.039	5.507	5.662

There was no significant interaction between the alcohol status of tweet (alcohol-related or unrelated to alcohol) and the pandemic period on *Personal Concerns* (F(1, 77) = 0.682, p = 0.411) or between the alcohol status and the total number of tweets (F(1, 77) = 0.417, p = 0.520).

The results show that personal concerns were more prevalent in alcohol-related tweets, regardless of the pandemic period. This result suggests that alcohol may be used as a coping mechanism for the insecurities (e.g. financial, occupational, health) brought on by the emerging crisis, as well as accompanying references to other potential coping mechanisms (e.g. leisure, religion).

## Discussion

The social upheaval brought about by the COVID-19 pandemic has led to questions of how individuals have been coping with such dramatic and abrupt life changes. Of particular concern is that individuals will turn towards outlets such as alcohol which, if abused, can have negative physical and mental health outcomes. For example, prior research has shown that stressful situations can result in increased chances for substance abuse, including alcohol. By examining social media communication, this study intended to assess whether the presence of alcohol references in said communication aligned with different levels of emotional, psychological, and social sentiment, perhaps indicating that alcohol was increasingly used as coping mechanism during the onset of the pandemic.

The study results found that the pandemic onset had a significant influence on the psychological, social, and emotional sentiment of COVID-related Twitter communication. Tweets in the post-pandemic period–the period after the WHO pandemic declaration in March 2020 –contained more honest (authentic) language, more affiliation-oriented language, and exhibited a more positive emotional tone. The increased use of language reflecting honesty can be reasonably expected; crises such as the COVID-19 pandemic can have a focusing impact on priorities and communication styles [35] which increase the ability of individuals to express hope, fear, and other emotions. Along this line of reasoning, previous research has found that individuals experiencing the extreme impacts of the distal effects of the pandemic’s onset (loss of income, lower social connectedness, etc.) were more likely to report increased alcohol consumption in general as well as increased use of alcohol as a means of coping [63]. This helps to explain the increased themes of honesty for alcohol-related tweets above that of non-alcohol-related tweets, specifically that alcohol played an important (albeit unhealthy) role for those suffering the most from the COVID-19 crisis.

The positive emotional tone exhibited in post-pandemic tweets is consistent with prior research [41] which suggests that expressing positive emotions can act as a protective measure in times of crisis. In this instance, the use of positive emotion-related language may reflect the (then) popular narrative that the social upheaval caused by the pandemic would be short-lived. The mantra of “two weeks to flatten the curve” was common among public officials during the post-March 11, 2020 period, and governmental responses to the situation were still evolving.

These findings are also consistent with prior research [35] which suggests that affiliation-oriented references increase in the short-term following a crisis event. Catastrophes, whether it be immediate events such as tornadoes or more longitudinal crises such as war, have been shown to increase tendencies to strengthen social bonds or “come together” to endure the event. Prior research [35] has also suggested this ‘short-term impact” of increased affiliation references quickly returns to a pre-crisis normal; given the ongoing nature of the COVID-19 pandemic and related restrictions, future research should explore whether this increase in affiliation-related language–and positive emotional tone–persists over the long-term.

The study results found alcohol-related status to be a significant main effect factor in only one instance–when individuals tweeted about personal concerns (e.g., faith, money, work, leisure, death, and home)–and this difference was consistent across both periods (pre- and post-pandemic). Prior research [23, 24] has pointed towards alcohol as a coping strategy for severe disruptions (e.g., financial crises, natural disasters) and has been shown to comingle with declines in psychological health [25, 26]. The rapid onset of the COVID-19 pandemic meant that an obscure reference in news reports became an issue impacting every man, woman, and child on the planet in a matter of weeks. The rapidity of this change, coupled with the uncertainty (financial, social, health, and otherwise) caused by the associated disruptions, may have exacerbated the use of alcohol as a coping mechanism for personal concerns.

While alcohol-related status was not a significant factor alone in regard to the use of honest (authentic) language within these tweets, alcohol status was found to have a significant interaction with the pandemic period; authenticity levels increased significantly more in alcohol-related tweets during the post-pandemic period than in tweets with no alcohol references. While the overall findings suggest that alcohol may play a lesser role in the expression of psychological, social, and emotional sentiment than the pandemic period, the interaction between the prevalence of honest (authentic) language and alcohol references may reflect an increased use of alcohol as a means of coping. This method of coping with the new reality associated with the onset of the pandemic, may have helped to either facilitate open and honest expression–as opposed to more tentative or guarded expression–or perhaps enabled individuals to endure the sometimes painful process of “opening up” during a time of crisis.

## Limitations

There are three potential limitations associated with this research. First, the use of English-only tweets without geolocation limits the ability to generalize the findings to a specific area. The use of the pandemic declaration date (March 11, 2020) as a dividing line between the pre- and post-pandemic periods provides a common time reference, despite the differing impacts of the pandemic across nations. A second potential limitation is the comparatively short timeframe for data collection (80 days). This timeframe was based on the accessibility of publicly available data due to Twitter API restrictions and provides a balance between the emergence of the pandemic and its immediate impacts. Finally, it must be noted that references to alcohol within tweets may not reflect patterns in use. However, alcohol references may indicate a willingness to use alcohol and, thus, be predictive of future behavior. Prior research has supported using social media to predict behaviors, including but not limited to alcohol consumption (e.g., [28, 64, 65]).

## Conclusion

In conclusion, the current study represents, to our knowledge, the only investigation into alcohol use as a coping mechanism during the onset of the COVID-19 pandemic using content analysis of alcohol- related tweets. Social media outlets like Twitter can act as a lens by which individuals express both psychological characteristics as well as coping behaviors during crises, and the study results show differences in in the expression of psychological, social, and emotional sentiment based on time period (pre- and post-pandemic) and, in one instance, the presence of alcohol references. The results found that alcohol-related tweets following the onset of the pandemic did not reflect changes related to confidence, emotional tone, affiliation references, or personal concerns compared to non-alcohol associated tweets. However, alcohol-related tweets during the post-pandemic period did reflect a greater degree of authenticity/honesty. This result may reflect alcohol use as a means of coping with the abrupt and dramatic changes associated with the pandemic. Specifically, in alcohol- related tweets individuals appeared to use more candid language following the initial onset of the pandemic. Alcohol-related tweets were also more likely to include content related to personal concerns regardless of the pandemic period, providing potential further indication of alcohol use as a coping mechanism. Future research should examine whether alcohol use for coping has become more prevalent as the pandemic has continued to upset societal norms.
